# BrainAGE in superagers: cross-sectional and longitudinal analyses in older adults aged 80+ with youthful episodic memory

**DOI:** 10.1007/s11357-025-01836-x

**Published:** 2025-08-16

**Authors:** Christian Gaser, Marta Garo-Pascual, Bryan A. Strange

**Affiliations:** 1 Structural Brain Mapping Group, Department of Neurology, Jena University Hospital, Jena, Germany; 2 Department of Psychiatry and Psychotherapy, Jena University Hospital, Jena, Germany; 3 German Centre for Mental Health (DZPG), Site Jena-Magdeburg-Halle, Magdeburg, Germany; 4 Laboratory for Clinical Neuroscience, Centre for Biomedical Technology, Universidad Politécnica de Madrid, IdISSC, Madrid, Spain; 5 Alzheimer Disease Research Unit, CIEN Foundation, Queen Sofia Foundation Alzheimer Centre, Madrid, Spain

**Keywords:** Superager, Episodic memory, Brain age, Neuroimage

## Abstract

Episodic memory, the ability to recall past events, is particularly vulnerable to ageing. A decline in episodic memory performance is generally considered part of ageing. However, the episodic memory performance of superagers —defined as individuals aged 80+ years old with episodic memory of people 30 years younger— is superior to that typical of their chronological age. The aim of this study was to determine whether the discrepancy between the superager's episodic memory and chronological age is also evident in their brain age. A BrainAGE (Brain Age Gap Estimation) approach, a multidimensional computational neuroanatomical method that uses structural neuroimaging data to estimate biological brain age, was applied. The study population comprised 64 superagers (mean age = 81.9 ± 1.9) and 55 age-matched typical older adults (82.4 ± 1.9). Cross-sectional analyses revealed a negative BrainAGE score for superagers (mean = -0.95 ± 2.36) indicating a deceleration of the ageing process. By contrast, typical older adults showed an average score close to zero (0.05 ± 3.03) consistent with their chronological age. The BrainAGE score of superagers was found to be lower relative to typical older adults, and the progression over a 5-year follow-up period was slower in superagers, in keeping with their youthful memory ability. Therefore, superagers have a younger brain than those of typical older adults, suggesting that their ageing mechanisms may involve resistance to age-related brain structural changes. However, despite a 30-year gap in episodic memory, their BrainAGE score differed by only one year, indicating that factors beyond brain structure contribute to the superager phenotype.

**Graphical Abstract:**

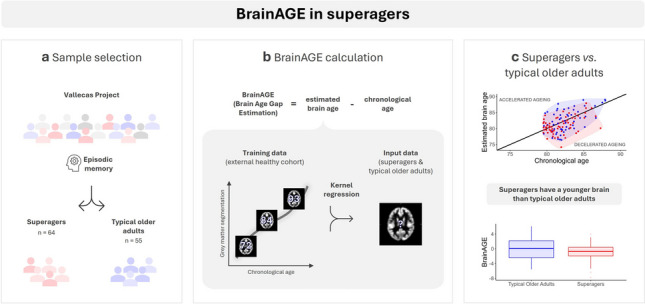

## Introduction

Chronological age is a measure of calendar years. However, it does not necessarily reflect the accumulation of changes that may or may not occur as part of the physiological ageing processes. The idea of biological age has been raised as a better proxy of age-related changes [1, 2] and has been conceptualise in many forms, including epigenetic [3, 4], telomeric [5], metabolic [6], or transcriptomic markers [7] and organ-specific clocks such as the brain or the heart [8, 9], among others.

As our interest is to understand the ageing mechanism of superagers, an elderly population with the episodic memory function of individuals 30 years younger [10–12], we focused on their brain age. The most extended brain age estimations are derived from brain imaging data and yield a single value that captures the complex multidimensional pattern of the brain [13]. The prediction models for brain age are trained on neuroimaging datasets from healthy individuals. These are pre-processed and down-sample to prevent over-fitting. Subsequently, kernel regression techniques are then applied to automatically select the relevant features of the multidimensional pattern of the brain. From this model of brain age, individual brain ages can be estimated. This approach has demonstrated to be sensitive in detecting brain changes associated with developmental, healthy ageing and neurodegenerative processes [14]. Brain age has been shown to predict the conversion from mild cognitive impairment to Alzheimer’s disease [15] and has also shown an association with cognitive functions in healthy ageing populations, more consistently with psychomotor speed but also other cognitive functions such as semantic verbal fluency, visual attention, or cognitive flexibility [16–18].

The brains of superagers exhibit a reduced atrophy rate in global grey matter terms, including both grey matter volume [12] and cortical volume [19]. In particular, a slower grey matter atrophy rate has been observed in the medial temporal lobe of superagers relative to age-matched peers [12]. A younger cohort of superagers (72.7 years on average) showed a smaller brain age relative to typical older adults [20]. The goal of estimating the brain age in a cohort of superagers over 80 years old is to determine whether their brains are younger later in life than their peers with normal memory for their age.

In this study, the BrainAGE (Brain Age Gap Estimation) approach was implemented, defined as the gap between the estimated brain age derived from the application of kernel regression methods to structural MRI (magnetic resonance imaging) data [14, 21] and chronological age. Positive values indicate accelerated ageing, and negative values indicate decelerated ageing. BrainAGE was calculated in 64 superagers and 55 typical older adults from the Vallecas Project cohort [12, 22]. The scores were compared between the two groups both cross-sectionally and longitudinally over a 5-year follow-up period.

## Methods

### Participants

In this study, the sample of superagers and typical older adults was drawn from the single-center, community-based Vallecas Project, a longitudinal cohort in Madrid, Spain. The Vallecas Project comprises 1,213 participants, all Caucasian ethnicity, aged 70 to 85 years at recruitment, living independently, with a survival expectancy of at least four years, and free from neurological or psychiatric disorders [22]. All participants provided written informed consent, and the project received approval from the Ethics Committee of the Instituto de Salud Carlos III.

We defined superagers as individuals aged 80 years or older with episodic memory performance equivalent to that of individuals 30 years younger [10–12]. Criteria for this analysis included age, episodic memory performance, cognitive performance in non-memory domains, stability of episodic memory and MRI availability. Both superagers and typical older adults were at least 79.5 years old when their episodic memory was assessed using the free delayed recall score on the Spanish Free and Cued Selective Reminding Test. Superagers were required to perform at or above the mean score of adults aged 50–56 years with similar educational attainment, while typical older adults scored within one standard deviation of the normative values for their age and education attainment according to the Spanish NEURONORMA project [23]. Further details on the selection process for superagers and typical older adults from the Vallecas Project were published previously [12].

### MRI images acquisition

MRI images were acquired using a 3 Tesla MRI (Sigma HDxt GEHC, Waukesha, USA) with a phased array 8 channel head coil. T1-weighted images (3D fast spoiled gradient echo with inversion recovery preparation) were collected using a TR of 10 ms, TE of 4.5 ms, FOV of 240 mm and a matrix size of 288 × 288 with slice thickness of 1 mm, yielding a voxel size of 0.5 × 0.5 × 1 mm.

### BrainAGE estimation

The MRI data were preprocessed using CAT12.9 [24], focusing on grey matter segmentation. These segmentations were then affinely registered and resampled to both 4 mm and 8 mm and smoothed with both 4 mm and 8 mm full-width half-maximum (FWHM) Gaussian kernel. To estimate BrainAGE, we used a training dataset of 601 subjects ranging in age from 70.0 to 97.2 years, with a mean age of 76.4 (standard deviation (SD) = 4.9) years, consisting of 294 males and 307 females. The resulting four BrainAGE estimates were integrated using a weighted average approach, with weights assigned according to the squared mean absolute error (MAE) of each model. In addition, a linear trend correction was applied to adjust for any age-related bias to ensure more accurate and reliable BrainAGE estimates. The MAE of the resulting brain age prediction model is 2.40 years.

### Statistical analysis

Before conducting cross-sectional group comparisons, the normality of the variables was assessed by visual inspection with Q-Q plots and by the Shapiro–Wilk normality test. Chi-squared tests and Fisher’s exact tests were used for comparisons of categorical data and two-sample t-tests and Mann–Whitney U test (two-tailed) were used for continuous variables with significance level set at 0.05.

Longitudinal trajectories of BrainAGE were studied with a linear mixed effects model built with the lme4 package in R [25] where BrainAGE score was fitted with scaled, but not centred, chronological age, group and the interaction between scaled chronological age and group as fixed factors together with the random intercept and the random slope.

All analyses were performed using R version 4.1.2 (https://www.r-project.org/).

## Results

**Fig. 1:**
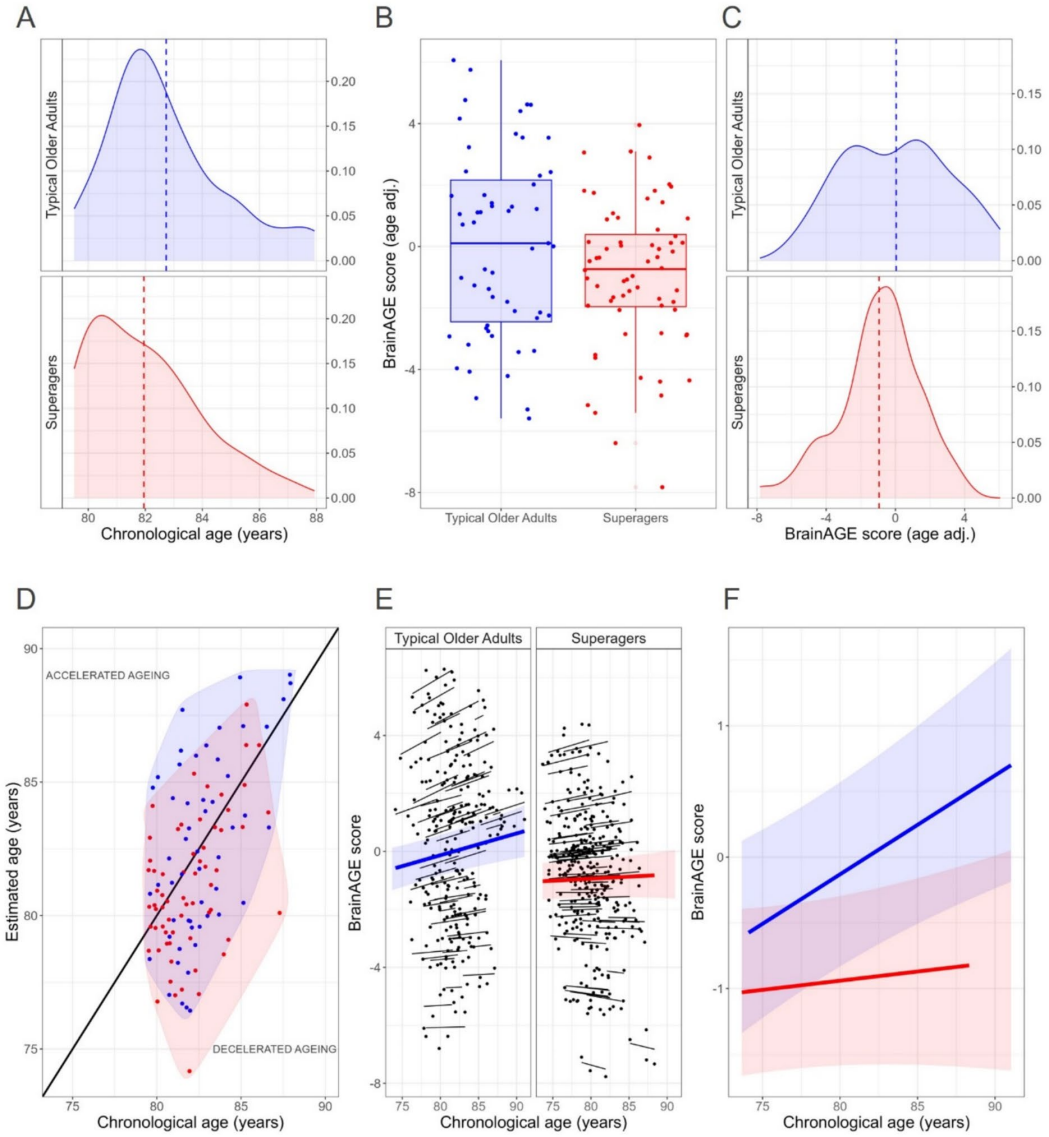
BrainAGE in superagers and typical older adults.** A** Difference in chronological age distribution between superagers and typical older adults. **B** Cross-sectional BrainAGE scores adjusted for chronological age presented as box and whisker plots and **C** density plots show a significantly smaller score in superagers relative to typical older adults (dashed line represents group means). **D** Comparison of chronological age and estimated brain age reveals whether an individual has accelerated or decelerated brain ageing. The red point cloud indicates that superagers have a greater tendency to decelerated brain ageing than typical older adults (blue cloud). **E** Individual longitudinal evolution of BrainAGE scores (black lines) and **(F)** group predictions illustrate a significant group-by-chronological-age interaction indicating a faster brain ageing in typical older adults (blue) than in superagers (red). The shaded areas represent the 95% confidence interval

Superagers and typical older adults were matched for chronological age. Although there were no significant differences in the group means (Table 1), the distribution differed between them (Fig. 1A). Therefore, BrainAGE was adjusted for chronological age to conduct the cross-sectional group comparisons. Adjusted BrainAGE was found to be significantly smaller in superagers than in typical older adults (Table 1, Fig. 1B-C). While superagers exhibited a negative mean BrainAGE score, indicating decelerated brain ageing on average, typical older adults displayed an average score close to zero, indicating that their brain is ageing at the pace it is supposed to by their chronological age (Fig. 1D).

**Table 1:** Cross-sectional characteristics of superagers and typical older adults

	Superagers (*n* = 64)	Typical older adults (*n* = 55)	Statistic	*P*-value
*Demographics*				
** Age**, mean (SD), years	81.9 (1.9)	82.4 (1.9)	Z = −1.8	0.08
** Women**, No. (%)	38 (59)	35 (64)	X = −0.1	0.77
** Education**, mean (SD), years	14.6 (6.0)	11.7 (7.2)	Z = 2.4	0.02
*Neuropsychology – selection criteria variables*				
** Free Cued Selective Reminding Test (free delayed recall)**, mean (SD)	13.4 (1.4)	6.5 (1.6)	Z = 9.4	< 2 × 10^–16^
** Semantic Fluency Test (animals)**, mean (SD)	21.2 (4.8)	15.9 (4.1)	t = 6.5	2 × 10^–9^
** Digit Symbol Substitution Test**, mean (SD)	21.2 (6.1)	15.3 (5.8)	t = 5.4	4 × 10^–7^
** 15-Boston Naming Test**, mean (SD)	13.8 (1.4)	11.5 (2.5)	Z = 5.4	7 × 10^–8^
*BrainAGE score*				
** BrainAGE score (age adjusted)**, mean (SD)	−0.95 (2.36)	0.05 (3.03)	t = −1.97	0.05

Longitudinal examination of BrainAGE, which involved MRI scans acquired in equivalent yearly follow-up visits from both superagers and typical older adults (superagers: mean (SD) visit number 5.17 (1.05); typical older adults: mean (SD) visit number 5.05 (1.35)), revealed a significant group-by-chronological-age interaction (b (se): −4.99 (2.30), *P* = 0.03). Typical older adults showed a faster increase in BrainAGE across time relative to superagers (slope (se), BrainAGE unit/one standard deviation of scaled age: superager: 1.0 (2.1); typical older adult: 5.4 (2.4)) (Fig. 1E-F) indicating that the brain of superagers is ageing at a slower rate compared to typical older adults.

## Discussion

The BrainAGE method provides a single value that captures the multidimensional ageing patterns present within brain structural MRI images. At the cross-sectional level, superagers exhibited a significantly lower BrainAGE score in comparison to typical older adults. The negative average of BrainAGE score in superagers at cross-sectional level is indicative of deceleration in their brain ageing. This finding dovetails with the significantly slower BrainAGE load over time in superagers compared to typical older adults. Therefore, using this age-specific method, superagers over 80 have an overall younger brain than typical older adults.

The most plausible is that superagers represent an extreme form of normal ageing outside of the pathological spectrum, as evidenced by the levels of blood biomarkers associated with neurodegeneration [12, 26–28]. Extending the terminology of Alzheimer’s disease [29] to the context of normal ageing, resistance refers to the avoidance of the effects of ageing and, in terms of brain age, will translate into a deceleration of the ageing process. Resilience, on the contrary, is the ability to cope with age-related changes and will translate into a similar estimated brain age and chronological age. The results of this study support the idea that the potential mechanism underlying grey matter ageing in superagers is resistance to age-related changes [12].

Given that superager's episodic memory performance is equivalent to people in their 50s, and typical older adults perform average for their 80s, it is reasonable to expect a large magnitude in BrainAGE differences. The differences in BrainAGE between the groups are significant cross-sectionally and longitudinally, however, the cross-sectional differences in BrainAGE between groups is ~ 1 year. There are a number of possible explanations for the magnitude of this difference, which are not mutually exclusive.

On the one hand, superagers are elders defined by excellent episodic memory for their age, but it is debatable whether the superager phenotype recapitulates a holistic approach on healthy ageing. Superagers outperform typical older adults on non-memory tests, thus they also excel in other cognitive domains other than episodic memory [12, 26, 30, 31]. Beyond cognition, there is also evidence that superagers have better mobility and better mental health, less hypertension and hyperglycaemia, or more satisfying social relationships [12, 32]. However, it remains unclear whether these attributes associated with superageing are common to all superagers, or whether the superager group is heterogeneous. In the case of a heterogeneous group, the fact that all attributes of superagers (in the cognitive, mental, motor or social domain, etc.) do not converge in the same person could explain the discrepancy between the expected and the observed magnitude of differences in BrainAGE.

On the other hand, the modest BrainAGE differences between groups might be attributable to a relatively late emergence of brain structural differences in superagers. By the age of 75, five years prior to the age at which individuals could be classified as superagers or typical older adults, total grey matter volume and ROI-based white matter microstructural properties including fractional anisotropy and mean diffusivity were indistinguishable between the superager and the typical older adult group reported here, despite the already existing differences in episodic memory performance [12, 33]. It is possible that functional differences precede structural ones. Consequently, future research that combines the structural brain clock currently employed with other functional brain clocks [34] might show a larger magnitude of the effect.

## Data Availability

The anonymised Vallecas Project data collection can be accessed upon request at direccioncientifica@fundacioncien.es.
